# The good, the bad, and the hazardous: comparative genomic analysis unveils cell wall features in the pathogen *Candidozyma auris* typical for both baker’s yeast and *Candida*

**DOI:** 10.1093/femsyr/foae039

**Published:** 2024-12-04

**Authors:** María Alvarado, Jesús A Gómez-Navajas, María Teresa Blázquez-Muñoz, Emilia Gómez-Molero, Sebastián Fernández-Sánchez, Elena Eraso, Carol A Munro, Eulogio Valentín, Estibaliz Mateo, Piet W J de Groot

**Affiliations:** Institute for Biomedicine, ETSIAMB, University of Castilla-La Mancha, 02008 Albacete, Spain; Institute for Biomedicine, ETSIAMB, University of Castilla-La Mancha, 02008 Albacete, Spain; Institute for Biomedicine, ETSIAMB, University of Castilla-La Mancha, 02008 Albacete, Spain; Institute for Biomedicine, ETSIAMB, University of Castilla-La Mancha, 02008 Albacete, Spain; Institute for Biomedicine, ETSIAMB, University of Castilla-La Mancha, 02008 Albacete, Spain; Department of Immunology, Microbiology and Parasitology, Faculty of Medicine and Nursing, University of the Basque Country (UPV/EHU), 48940 Bilbao, Spain; Institute of Medical Sciences, University of Aberdeen, Aberdeen AB25 2ZD, United Kingdom; GMCA Research Unit, Departament of Microbiology and Ecology, University of Valencia, Burjassot, 46010 Valencia, Spain; Severe Infection Research Group, Health Research Institute La Fe, 46026 Valencia, Spain; Department of Immunology, Microbiology and Parasitology, Faculty of Medicine and Nursing, University of the Basque Country (UPV/EHU), 48940 Bilbao, Spain; Institute for Biomedicine, ETSIAMB, University of Castilla-La Mancha, 02008 Albacete, Spain

**Keywords:** *Candida auris*, cell wall, candidiasis, adhesins, GPI proteins, glucan, mannosylation

## Abstract

The drug-resistant pathogenic yeast *Candidozyma auris* (formerly named *Candida auris*) is considered a critical health problem of global importance. As the cell wall plays a crucial role in pathobiology, here we performed a detailed bioinformatic analysis of its biosynthesis in *C. auris* and related *Candidozyma haemuli* complex species using *Candida albicans* and *Saccharomyces cerevisiae* as references. Our data indicate that the cell wall architecture described for these reference yeasts is largely conserved in *Candidozyma* spp.; however, expansions or reductions in gene families point to subtle alterations, particularly with respect to β­-1,3-­glucan synthesis and remodeling, phosphomannosylation, β-mannosylation, and glycosylphosphatidylinositol (GPI) proteins. In several aspects, *C. auris* holds a position in between *C. albicans* and *S. cerevisiae*, consistent with being classified in a separate genus. Strikingly, among the identified putative GPI proteins in *C. auris* are adhesins typical for both *Candida* (Als and Hyr/Iff) and *Saccharomyces* (Flo11 and Flo5-like flocculins). Further, 26 putative *C. auris* GPI proteins lack homologs in *Candida* genus species. Phenotypic analysis of one such gene, *QG37_05701*, showed mild phenotypes implicating a role associated with cell wall β-1,3-glucan. Altogether, our study uncovered a wealth of information relevant for the pathogenicity of *C. auris* as well as targets for follow-up studies.

## Teaser

A comparative genomic analysis of cell wall synthesis in the pathogenic yeast *C. auris*.

## Introduction

Fungal bloodstream infections in humans are primarily caused by budding yeasts commonly known as *Candida* spp. to which humans are frequently exposed and that are often present in the human body as commensal members of the microbiota. Most frequent manifestations of candidiasis are cutaneous and mucosal infections, however, the more problematic are life-threatening invasive infections, including candidemia. Each year, there are ~400 000 cases of candidemia worldwide, with mortality rates surpassing 40% (Koehler et al. 2019, World Health Organization 2022, Denning 2024).

**Figure 1.**
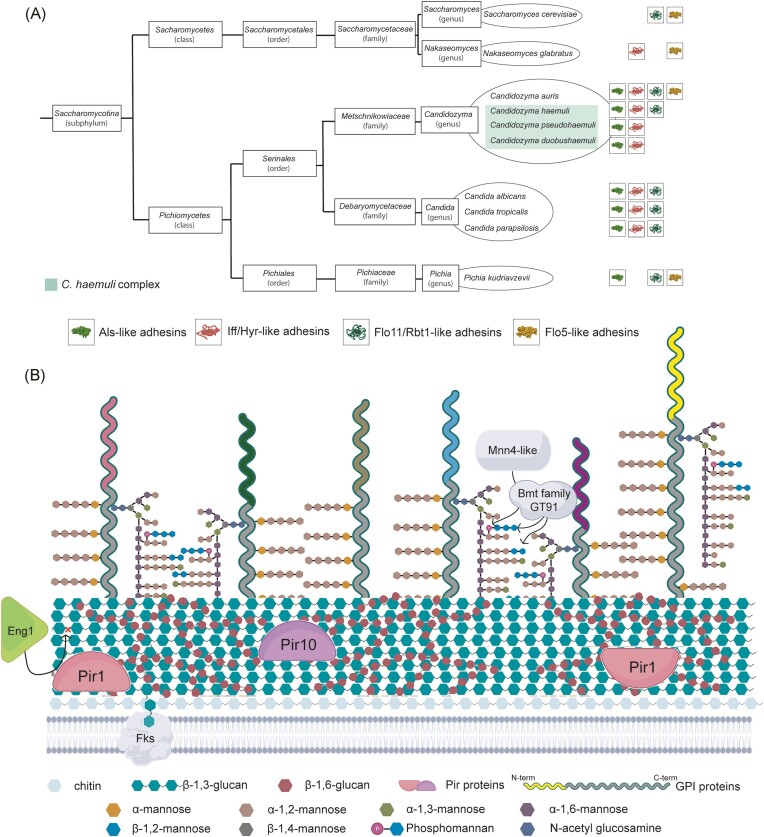
Genomic analysis of cell wall biosynthesis in *C. auris*. (A) Taxonomic tree including the species analyzed in this study and the most important causal agents of candidiasis according to the WHO (World Health Organization 2022). Putative adhesin families present in each of the species are indicated on the right. (B) Proposed cell wall architectural model based on genomic data. Major distinctive features explained in the text are indicated. Colors of N-terminal parts of GPI proteins represent different protein families (including adhesins).

The original genus *Candida* comprises ~200 different species, but only about a dozen are considered significant causal agents of human pathologies (Pfaller et al. 2019, Lass-Florl et al. 2024). The taxonomy of the causal agents of candidiasis is currently undergoing some major changes, including reassignment of genera and species names of some of the major pathogenic species such as *Candida glabrata* (renamed *Nakaseomyces glabratus*), *Candida krusei* (renamed *Pichia kudriavzevii*), and *Candida auris* (renamed *Candidozyma auris*) (Borman and Johnson 2023, Liu et al. 2024, Spruijtenburg et al. 2024) (Fig. 1A). The group of *Candida* (and other) species that translate CTG codons predominantly to serine instead of leucine residues are now classified in the order *Serinales*. The new names are in better accordance with phylogenomic data although for *N. glabratus* and *P. kudriavzevii* one could argue that they do not reflect being important causal agents of candidiasis, for simplicity hereafter referred to as *Candida* species.

Recently, the drug-resistant yeast *C. auris* has emerged as a species with a high incidence in clinical settings, making it one of the most prevalent candidiasis-causing species in certain hospitals (Lockhart et al. 2023, De Gaetano et al. 2024). *C. auris* impacts at-risk populations, including the elderly, critically ill, and immunosuppressed individuals, causing candidemia and other severe infections, resulting in mortality rates approaching 60% (Chakrabarti et al. 2015, Arensman et al. 2020, Hata et al. 2020, World Health Organization 2022). Its attributes include persistence in hospital environments and care homes, easy transmission, and high drug resistance, in most cases to fluconazole but in some cases to polyenes and/or echinocandins (Arendrup et al. 2017, Chowdhary et al. 2017, Lockhart et al. 2017, Centers for Disease Control and Prevention 2024, Lockhart et al. 2023). Altogether, this makes *C. auris* a serious health threat worldwide, and explains why it has been marked as a “Critical Pathogen” on WHO’s Fungal Priority Pathogens list (World Health Organization 2022).

*C. auris* is a haploid yeast belonging to the order *Serinales* (Fig. 1A). It appears to have a highly plastic karyotype as strains can undergo rapid stress-induced karyotypic changes (Bravo Ruiz et al. 2019, Narayanan et al. 2022). *C. auris* isolates have been classified into four main genetically distinct geographical clades [South Asian (I), East Asian (II), African (III), and South American (IV)] exhibiting different pathogenic characteristics. New clades from Iran and Bangladesh, and Singapore have subsequently been identified, taking the total to six (Lockhart et al. 2017, Casadevall et al. 2019, Chow et al. 2019, Rhodes and Fisher 2019, Khan et al. 2024, Suphavilai et al. 2024). While isolates belonging to clades I, III, and IV show a relatively high frequency of multidrug resistance and cause outbreaks that are difficult to control, clade II is predominantly associated with ear infections and appears to be less resistant to antifungals (Welsh et al. 2019).

Like other *Candida* spp., *C. auris* is capable of biofilm formation, morphological transition, and phenotypic variation, which contributes to its virulence and survival (Borman et al. 2016, Sherry et al. 2017, Yue et al. 2018, Singh et al. 2019, Horton et al. 2023). It endures on human skin and surfaces for weeks, survives desiccation, and withstands exposure to some disinfectants, promoting its hospital outbreak resilience (Welsh et al. 2017, Eyre et al. 2018, World Health Organization 2022).

By playing a pivotal role in surface contact and primary host-pathogen interactions, the *Candida* cell wall is of key importance for the various virulence mechanisms mentioned (Klis et al. 2009, Gow et al. 2017). In related yeast species like *Candida albicans*, the inner part of the cell wall is predominantly composed of a carbohydrate meshwork containing β-1,3-glucan, β-1,6-glucan, and chitin. In contrast, the outer part is densely packed with mannosylated proteins, the majority being glycosylphosphatidylinositol (GPI)-modified proteins that are covalently bound to β-1,6-glucan molecules. These proteins, mostly members of paralog families, have a multitude of different functions such as cell wall maturation and modification, breakdown and utilization of (host) substrates in the surrounding environment, and facilitation of surface adhesion and biofilm formation (De Groot et al. 2005). Notably, genomic investigations have revealed an enrichment of certain GPI protein families such as Hyr/Iff adhesins in pathogenic *Candida* species compared to their nonpathogenic counterparts, suggesting a direct link to pathogenicity (Plaine et al. 2008, Butler et al. 2009). Apart from covalently bound cell wall proteins (CWPs), recent studies have also documented the presence of noncovalently bound proteins, postulated to travel to the cell surface as cargo of extracellular vesicles (Gil-Bona et al. 2015). Most of those proteins are known as abundant cytosolic proteins with known functions, and whether they play a role at the cell surface is still a matter of debate.

GPI-modified adhesins provide yeasts with the capacity to adhere to diverse substrates like host cells or abiotic surfaces such as catheters and other medical devices. This adherence capacity is crucial in fungal colonization and establishment of infections (Sundstrom 2002, Tronchin et al. 2008). Moreover, fungal cell-to-cell adhesion promotes the creation of biofilms embedded in an extracellular matrix that supports resistance to antifungal agents (d'Enfert 2006, Gulati and Nobile 2016, Costa-Orlandi et al. 2017). Genomic analysis revealed that *C. albicans* encodes for three GPI protein families with demonstrated or presumed adhesive functions (De Groot et al. 2013): Als (Hoyer et al. 2008), Hyr/Iff (Richard and Plaine 2007), and Hwp (Hayek et al. 2010), conserved across other *Debaryomycetaceae* (Butler et al. 2009). Interestingly, in a seminal paper by Santana and colleagues, the Scf1 GPI adhesin in *C. auris*, whose N-terminal domain shows structural similarity to the Flo11 flocculin of *Saccharomyces cerevisiae*, was shown to be required for biofilm formation, skin colonization, virulence, and colonization of inserted medical devices (Santana et al. 2023).

Besides adhering to host cells, *Candida* species secrete hydrolytic enzymes that destabilize or degrade host membranes and proteins, facilitating tissue penetration and enhancing extracellular nutrient acquisition efficiency (Wachtler et al. 2012, Bras et al. 2024). Three classes of such secreted or cell wall-associated hydrolases are described in *Candida* species: aspartic proteases (Naglik et al. 2003), phospholipases (Niewerth and Korting 2001), and lipases (Hube et al. 2000). Some of the aspartic proteases and phospholipases appear to be retained in the cell wall through GPI anchoring (Niewerth and Korting 2001, Butler et al. 2009, Schild et al. 2011). The expansion of genes encoding aspartic proteases in pathogenic species compared to less pathogenic relatives supports a role for these proteins in the infection process (Butler et al. 2009, Moran et al. 2012).

Here, aiming to improve our understanding of how cell wall genomics impacts on pathobiology of *C. auris*, we performed an extensive bioinformatic analysis of the cell wall biosynthetic machinery in different strains, including the reference genomes from all six clades. Comparison to *S. cerevisiae* and *C. albicans* indicated that the overall architecture of the cell wall was conserved, which is consistent with earlier observations based on fluorescence staining (Shivarathri et al. 2020). Importantly, *C. auris* seems to take an intermediate position regarding some cell wall characteristics that are distinct when compared to *S. cerevisiae* and *C. albicans*. Further, the inclusion of related species of the *Candidozyma haemuli* complex in our genomic analysis allowed us to pinpoint several genes that may be relevant for the elevated pathogenicity of this critical pathogen.

## Materials and methods

### Genome sequence retrieval

Genome sequence assemblies of *C. auris* strains and *C. haemuli*-complex species were obtained from the National Center for Biotechnology Information (NCBI) (https://www.ncbi.nlm.nih.gov/genome/). Analyzed *C. auris* genomes included strains from all genetically distinctive clades: clade I strain VPCI479/P/13 and B11205 (IN) and B13916 (AE); clade II strain B11220 (JP), B11809 (KR), and B13463 (CA); clade III strain B11221 (ZA), B17721 and B12631 (US), and B12037 (CA); clade IV strain B11245 and B11243 (VE), and B12342 (CO); clade V strain IFRC2087 (IR); and clade VI strain F0083 (SG) (Sharma et al. 2015, Lockhart et al. 2017, Muñoz et al. 2018, Muñoz et al. 2021, Suphavilai et al. 2024). Analysis of *C. haemuli*-complex species was focused on genome assemblies of reference strains *C. haemuli* strain B11899, *C. pseudohaemuli* strain B12108, and *C. duobushaemuli* strain B09383 (Muñoz et al. 2018). For sequenced genomes lacking annotations in NCBI, gene predictions and translations were performed using Augustus 3.5.0 (https://github.com/Gaius-Augustus/Augustus) followed by manual checking and curation. Genome synteny between *C. auris* reference strains was analyzed using the NCBI comparative genome viewer tool.

### *In silico* analysis

To identify and annotate protein-encoding genes involved in cell wall biosynthesis, homology searches were performed with a local BLAST tool downloaded from EMBOSS (http://emboss.sourceforge.net/), using known fungal cell wall-related protein sequences as queries. As detailed in (Alvarado et al. 2023), homologs identified by Blast were judged by probability (E) values, identity levels over the whole sequence (in most cases >30%), and the presence of known functional domains. Blast hits were validated by cross-blast analysis.

In addition, an independent search for putative GPI proteins was performed using a previously described bioinformatic pipeline that selects proteins for the presence or absence of protein features in the primary sequences of the protein precursors (De Groot and Brandt 2012). (i) For the prediction of C-terminal GPI anchoring (“Predicted GPI proteins”), the fungal-specific algorithm Big-PI was employed (http://mendel.imp.ac.at/gpi/fungi_server.html, (Eisenhaber et al. 2004)). Big-PI has good specificity but is too strict (Butler et al. 2009, De Groot and Brandt 2012, Alvarado et al. 2023). Therefore, to include proteins with ambiguous GPI-anchoring features (“Ambiguous GPI proteins”), it was complemented with a more inclusive complementary approach based on pattern and composition scanning, performed using ProFASTA (De Groot and Brandt 2012). (ii) The presence of N-terminal signal peptides for secretion was determined with SignalP (https://services.healthtech.dtu.dk/services/SignalP-4.1/), and (iii) absence of internal transmembrane domains was analyzed using TMHMM (https://services.healthtech.dtu.dk/services/TMHMM-2.0/). Parsing of Big-PI, SignalP, and TMHMM data was performed with ProFASTA (De Groot and Brandt 2012). Proteins missed by the pipeline in some of the strains due to possible sequence or annotation errors were identified by Blast and subsequent manual inspection of the gene locus. Finally, all GPI protein candidates were subjected to NCBI Blast analysis for annotation purposes and removal of potential false positives with homology to non-GPI proteins.

Putative functional domains of GPI proteins showing weak primary sequence homology to adhesins or flocculins in *C. albicans* or *S. cerevisiae* were subjected to three-dimensional (3D) protein structure modeling using the AlphaFold2 algorithm (Mirdita et al. 2022), followed by homology searching using the Dali server (Holm 2020) and subsequent pairwise structure comparison at RCSB (https://www.rcsb.org/alignment). If no Protein Data Bank (PDB) entry was available for the weak homolog, its 3D structure was also modeled and aligned to verify that the primary sequence homology translates into tertiary structure relatedness. The sequence logo for the Flo5 42-aa repeat was prepared with WebLogo 3.7.12 (https://weblogo.berkeley.edu/logo.cgi) based on alignment of the repeats present in strains VPCI479/P/13, B11220, B11221, B11245, IFRC2087, and F0083. For the prediction of β-aggregation, we used TANGO (http://tango.crg.es) with default settings. 3D protein structure modeling and analysis were also performed for the aspartic protease Apr10 and Pir proteins.

If not already annotated differently, cell wall-related *C. auris* proteins identified in this study were tentatively named after their closest homologs in *C. albicans*, except for Flo5 which lacks a *C. albicans* homolog and was named after its closest *S. cerevisiae* homolog. *C. auris* proteins sharing the same closest homolog in *C. albicans* were named, in order of sequence identity, after the *C. albicans* query and then adding nominal numbers to the additional protein copies (e.g. Sap9, Sap90, Sap91, and Sap92).

### Gene expression analysis by real-time PCR

Gene expression of four predicted GPI protein-encoding genes in *C. auris* lacking paralogs in the *Candida* genus was measured by quantitative real-time PCR (qPCR). *C. auris* strain VPCI479/P/13 was grown in YPD at 37°C until the logarithmic phase in the absence or presence of subinhibitory concentrations (Minimal Inhibitory Concentrations, MIC_50_) of micafungin (Mic, 0.031 µg/ml), isavuconazole (Isa, 0.031 µg/ml), amphotericin B (Amb, 1.0 µg/ml), and Calcofluor white (CFW, 0.016 µg/ml), to stationary phase, and forming biofilms for 24 h on polystyrene Petri dishes. Cells were collected and broken (Fastprep-24), after which total RNA was isolated using TRIzol Reagent (Thermo Fisher Scientific) following standard procedures. cDNA was synthesized using 2 µg of RNA employing a High-Capacity RNA-to-cDNA kit.

For all genes, target genes as well as the 5.8S rDNA endogenous control, primer efficiency, and correct amplification were validated by generating standard curves and analysis on agarose gels. qPCR was performed using 4 µl of 20-fold (tested genes) or 100-fold (5.8S rDNA control) diluted cDNA in a 10 µl volume using Fast SYBR Green qPCR Master Mix and a 7500 Fast Real-Time PCR System (Applied Biosystems) following the manufacturer“s instructions. Ct values were used to calculate normalized expression levels against 5.8S rDNA, averaging values from two biological samples measured in triplicate. Primers used for qPCR are listed in Table S8.

### Generation of deletion mutants

*QG37_05701* and *QG37_01906*, two *Candidozyma*-specific GPI protein-encoding genes that were expressed under the tested conditions, were selected for phenotypic analysis. Deletion mutants were generated in strain VPCI479/P/13 using the *SAT1*-flipping system (Reuss et al. 2004), employing RNP-based CRISPR-Cas9 (Grahl et al. 2017) to achieve efficient integration into the correct genomic loci, as previously described (Reithofer et al. 2021). Deletion cassettes were removed from the genome by flippase-induced recombination of *FRT* sequences, and correctness of the deletion mutants was verified by PCR. Primers and CRISPR guides used are listed in Table S8. For both genes, two deletion mutants from independent transformation experiments were included as biological replicates in all phenotypic assays.

### Adhesion or biofilm formation onto polystyrene

Adhesion or biofilm formation on polystyrene was measured using two different assays depending on the time allowed to adhere (4 or 24 h). For the 24 h experiment, overnight pre-cultures in YPD (1% yeast extract, 2% peptone, 2% glucose) or Roswell Park Memorial Institute (RPMI) 1640 medium at 37°C were diluted to a cell density of OD_600_ = 0.5 (∼1 × 10^7^ cells/ml) in the respective fresh media, and 200 µl of the cell suspension was pipetted into a 96-well plate and incubated for 24 h at 37°C in a humid environment. Unattached cells were removed by gentle washing with mQ water, and the remaining cells that formed a biofilm were stained with 0.1% Crystal violet (CV) solution for 30 min followed by washing with mQ water. Finally, CV was solubilized in 33% glacial acetic acid and quantified by measuring the optical density values at 595 nm (OD_595_) using a microplate reader (Molecular Devices). Data for each strain are the average of four replicate cultures, each with six technical replicates.

For the 4 h adhesion experiment, overnight cultures were diluted to OD_600_ = 0.05 in phosphate-buffered saline (PBS), 0.5 ml was pipetted in 12-well plates and incubated for 4 h at 37°C. Unbound cells were removed by two washes with PBS, after which adhered cells were loosened and disaggregated by treatment with a 2.5% trypsin (from porcine pancreas, Sigma) in PBS solution for 10 min at room temperature (RT). Finally, the loosened cells were resuspended in a final volume of 0.5 ml PBS, and cell events were counted using a MACSQuant (Miltenyi Biotec) flow cytometer (Reithofer et al. 2021) Data are the average of two biological replicates measured in triplicate.

### Adhesion to HeLa cells

Adhesion to HeLa cells was determined as detailed in (Reithofer et al. 2021). Briefly, cells from overnight cultures (YPD, 37°C) were co-incubated with preformed confluent layers of HeLa cells for 2 h at 37°C with 5% CO_2_. Nonadhered cells were pipetted off and adhered cells were collected by scraping. Cells in both fractions were quantified by CFU counting after growth on YPD medium containing chloramphenicol (2 µg/ml) at 37°C. Controls without HeLa cells were included to discard fungal adhesion to plastic. Data shown are averages of at least two biological replicates measured in triplicate.

### Adhesion to extracellular compounds

Differences in the binding capacity to cell wall molecules or mammalian extracellular matrix collagen were evaluated by flow cytometry, as detailed in (Fernandez-Pereira et al. 2021). Briefly, microtiter plates were coated with pustulan (β-1,6-glucan), laminarin (β-1,3-glucan), chitin, or bovine collagen, washed with PBS, and incubated with 0.5 × 10^6^ overnight-grown cells diluted in 0.5 ml PBS. After 4 h of incubation at 37°C, adhered cells were loosened by treatment with trypsin and measured using a MacsQuant flow cytometer. Data shown are averages of four biological replicates measured in triplicate.

### Drug sensitivity assays

Susceptibility to antifungals and cell wall perturbants was tested in 96-well plates following EUCAST guidelines (J Guinea et al. 2023). Two-fold serial dilutions of compounds were prepared in YPD and mixed 1:1 with cells from overnight cultures diluted to an OD_600_ of 0.01. Plates were incubated for 24 h at 37°C. MIC were determined after reading the OD_600_ (two biological replicates, two technical replicates each) in a microplate reader. Compounds tested were Amb, fluconazole (Flu), Isa, caspofungin (Cas), Mic, CFW, and SDS.

Congo red (CR) sensitivity was determined using a drop assay. Ten-fold serial dilutions (two biological replicates) were prepared from overnight cultures adjusted to OD_600_ = 1, and 4 µl of each dilution were spotted on YPD plates containing 100 µg/ml CR. Growth was monitored after 24 and 48 h of incubation at 37°C.

### Growth rate and sensitivity to zymolyase

To evaluate the growth kinetics, overnight pre-cultures were diluted to an OD_600_ = 0.1 in fresh YPD medium, and 200 µl of cell suspensions were pipetted in a 96-well plate. Cells were incubated for 12 h at 37°C with agitation in a Spectra Max 340 plate reader (Molecular Devices), measuring the OD_600_ every 15 min. For determination of zymolyase sensitivity, cells were grown in YPD until log phase, collected, and resuspended in 10 mM Tris-HCl, pH 7.4 at an OD_600_ of 2.0 to which 0.25% of β-mercaptoethanol was added. After 1 h of incubation at RT, 180 µl of cell suspensions and 20 µl of 10 U/ml zymolyase were mixed in a 96-well plate and incubated at 37°C. Decrease in OD_600_ was measured every minute after a short mixing pulse. Curves represent averages of two biological and five technical replicates.

### Cell surface hydrophobicity and sedimentation

Cell surface hydrophobicity (CSH) was tested using the microbial adhesion to hydrocarbon test. Overnight-grown stationary phase and exponential phase cultures were washed twice with PBS and adjusted to an OD_600_ = 0.7. Cell suspensions were mixed with hexadecane in glass tubes at a 15:1 volume ratio. Upon 1 min of gentle vortexing, the phases were allowed to settle for 10 min after which the OD_600_ of the aqueous phase was measured. Each strain was assayed four times with two technical replicates each.

For analysis of sedimentation, overnight cultures in YPD at 37°C were transferred to 15 cm long glass tubes. For 1 h, sedimentation was monitored every 10 min by carefully taking 50 µl of sample from 2 cm beneath the surface for OD_600_ measurements. Each strain was assayed four times with three technical replicates each. Aggregation of the same cultures was monitored by flow cytometry.

### Virulence

Virulence of deletion mutants was tested using *Galleria mellonella* as *in vivo* invertebrate model of candidiasis. *Galleria mellonella* worms were purchased from Artroposfera (Toledo, Spain). Worms with a minimal size of 2 cm and showing no signs of melanization were selected and incubated at 37°C for 24 h to check their viability. Meanwhile, yeast pre-cultures were prepared by growing overnight in YPD at 37°C. These cultures were washed and diluted in PBS to a concentration of 5 × 10^6^ cells/ml. Ten µl of these suspensions were injected with a Hamilton syringe (Agilent Technologies, Madrid, Spain) into the last right pseudopod of the worms. Worms were incubated at 37°C, and survival was monitored daily for one week. Worms injected with PBS or *S. cerevisiae* (BY4741) were used as controls. Results shown are the mean of two independent blind experiments each performed with two biological replicates and groups of 15 *G. mellonella* worms per replicate.

### Statistical analysis

The statistical significance of phenotypic data was analyzed by Student's *t*-tests or one-way Analysis of Variance (ANOVA) followed by post hoc Delayed Matching to Sample tests. *P*-values < 0.05 were considered statistically significant.

## Results

To improve our understanding of the pathobiology of the drug-resistant yeast *C. auris*, we performed a detailed genomic-scale inventory and analysis of the genes involved in cell wall biogenesis, including genes that encode CWPs. Our analysis encompasses various *C. auris* isolates from all six distinct genetic clades. To identify possible differences between *C. auris* and phylogenetically closely related but clinically less relevant *Candida* species, the bioinformatic analysis was extended with reference genomes of three species of the *C. haemuli* complex *Candidozyma haemuli, Candidozyma pseudohaemuli*, and *Candidozyma duobushaemuli* (Fig. 1A).

With respect to cell wall biology, among the best-studied fungal species are *C. albicans* and *S. cerevisiae*. A similar cell wall architecture can be expected in *C. auris* and related species as they are phylogenetically related. To confirm this, the translated genomes of *C. auris* and *C. haemuli-*complex species were searched with known fungal cell wall biosynthetic proteins from *C. albicans* and *S. cerevisiae* as queries using a local Blast tool. These searches indeed identified orthologs for all these proteins (Table 1). To make the homology search as unbiased and inclusive as possible, proteins from less-related species, associated with the synthesis of other fungal cell wall macromolecules (e.g. α-1,3-glucan, mixed-linked β-1,3/1,4-glucans, and cellulose) not described in *Saccharomycotina*, were included as queries (Table S1). However, this did not yield additional cell wall biosynthetic genes, supporting the notion that the cell wall architecture described for *C. albicans* and *S. cerevisiae* is mostly conserved in *C. auris* and the *C. haemuli*-complex species.

**Table 1.** Comparative analysis of protein families involved in cell wall biosynthesis of *C. auris*.

Protein class^a^	*Cau* ^b^	*Ch*	*Cps*	*Cd*	*Cal*	*Sc*
***β-1,3-glucan synthesis and processing***						
Fks family. β-1,3-glucan synthases (GT48)	2	3	2	2	3	3
Rho1 family. Putative Rho-related GTPases	6	6	6	6	6	6
Putative regulatory component of β-1,3-glucan synthesis	2	2	2	2	2	1
Gas family. β-1,3-glucanosyltransglycosylases (GH72) involved in connecting the emerging β-1,3-glucan chains to the existing β-glucan network	5	5	5	5	5	5
Crh family. Transglycosylases (GH16) involved in crosslinking of β-glucan and chitin	3	3	3	3	3	3
Bgl2 family. Putative β-1,3-transglucosylases (GH17) involved in connecting β-1,3-glucan chains to the existing β-glucan network through β-1,6-linkages	4	4	4	4	5	4
Eng1-like endoglucanase (GH81)	3	3	3	3	2	2
Putative exo-β-1,3-glucanase family (GH5)	3	3	3	3	3	3
Sun family. Putative β-glucosidase activity, involved in septation (GH132)	2	2	2	2	2	4
Tos1 family. Predicted β-1,3-glucanase activity (GH16)	2	2	2	2	2	2
***Chitin synthesis and processing***						
Chitin synthases (GT2)	4	4	4	4	4	3
Chitinase (GH18); orthologs are sporulation-specific	2	2	2	2	4	2
Chitin deacetylase (CE4)	1	1	1	1	1	2
***Other proteins with putative functions in cell wall synthesis***						
Kre6-like putative transglycosylases (GH16) required for β-1,6-glucan biosynthesis	2	2	2	2	4	2
Dfg5 family. Endo-mannanases (GH76) involved in GPI anchor processing	2	2	2	2	2	2
Ecm33 family. Unresolved function (GH NC), possible role in CWP incorporation	3	3	3	3	3	4
Pir family. Possible role in β-1,3-glucan crosslinking. Contain multiple Pir repeats	2	2	2	2	1	5
***Protein mannosylation***						
Pmt family. Protein-O-mannosyltransferases (GT39)	5	5	5	5	5	7
Och1 family of α-1,6-mannosyltransferases (GT32). Initiate N-glycan outer chain branch addition	2	2	2	2	2	2
Anp1-like subunits of a Golgi α-1,6-mannosyltransferase complex (GT62)	3	3	3	3	3	3
Mnn10-like subunits of a Golgi α-1,6-mannosyltransferase complex (GT34)	2	2	2	2	2	2
Mnn4-like regulators of mannosylphosphorylation of N-linked mannans	6	6	6	6	8	2
Bmt family. β-1,2-mannosyltransferase (GT91). β-mannosylation of phosphopeptidomannan	2	2	2	2	9	0
ScKtr/CaMnt family of α-1,2-mannosyltransferases (GT15)	6	6	6	6	5	9
Mnn2 family of α-1,2-mannosyltransferases (GT71)	5	5	5	5	6	2
Mnn1 family of α-1,3-mannosyltransferases of Golgi complex (GT71)	6^c^	6	6	6	6	4

a Classification of carbohydrate-active enzymes according to CAZy Database (http://www.cazy.org/).

b *Cau, C. auris; Ch , C. haemuli; Cps , C. pseudohaemuli; Cd, C. duobushaemuli; Cal, C. albicans*; and *Sc, S. cerevisiae*.

c Five gene copies in clade II strain B11220.

The cell wall polysaccharides β-1,3-glucan and chitin are synthesized by plasma membrane-localized glycosyl transferase enzyme complexes. These proteins, as well as proteins with accessory or regulatory functions, such as the cell wall integrity pathway, and enzymes with polysaccharide-processing functions, are conserved in the analyzed *Candidozyma* species including *C. auris*. The number of gene copies of the respective families is also, in general, conserved. (Table 1, further detailed in Table S1) (Butler et al. 2009). Yet, the *Candidozyma* species analyzed (except *C. haemuli*) lack one of the three Fks family paralogs encoding β-1,3-glucan synthases (GT48) (Douglas 2001). Furthermore, an expansion from two to three paralogs was observed for the Eng1 family (GH81) described as endoglucanases involved in the modification or hydrolysis of β-1,3-glucan, particularly during processes such as cytokinesis (Esteban et al. 2005).

Although β-1,6-glucan in yeast cell walls is crucial for interconnecting β-1,3-glucan, chitin, and GPI-modified mannoproteins, its synthesis is poorly understood. For instance, an enzyme showing *in vitro* β-1,6-glucan-synthesizing activity remains unidentified to date though Kre6-like GH16 proteins have been postulated as candidates (Lesage and Bussey 2006, Ruiz-Herrera et al. 2006, Bekirian C. et al. 2024). *C. auris* harbors two Kre6 homologs in its genome similar to *S. cerevisiae*, contrasting with the four paralogs present in the phylogenetically closer *C. albicans*. The Ecm33 family, proposed to have an important but still unresolved role in CWP or β-1,6-glucan incorporation (Pardo et al. 2004, Chaabane et al. 2019), is represented by three members in *C. auris* and the *C. haemuli*-complex species. In *C. albicans* the Ecm33 family also has three members while *S. cerevisiae* possesses four paralogs (two twin pairs).

Proteins destined to be covalently attached to the polysaccharide network of the cell wall undergo significant glycosylation, mostly mannosylation, as they are translocated to the cell surface. Glycosylation takes place through two distinct processes: *O*-glycosylation and *N*-glycosylation, mediated by different families of mannosyltransferases. Blast searches with *S. cerevisiae* and *C. albicans* protein queries confirmed the presence of all families of mannosyltransferases in *C. auris* and the other species under study (Table 1 and Table  S1). Gene copy numbers were similar for those involved in α-mannosylation. Regarding gene families involved in N-glycosylation (Hall and Gow 2013), noteworthy is the presence of *C. auris* and *C. haemuli*-complex species of two Bmt paralogs responsible for β-mannosylation. This family is absent in *S. cerevisiae* but extensively expanded (nine proteins) in *C. albicans*. Also, the *Candidozyma* species contain six Mnn4 (GT34) proteins involved in phosphomannan addition compared to eight and two paralogs in *C. albicans* and *S. cerevisiae*, respectively.

**Figure 2.**
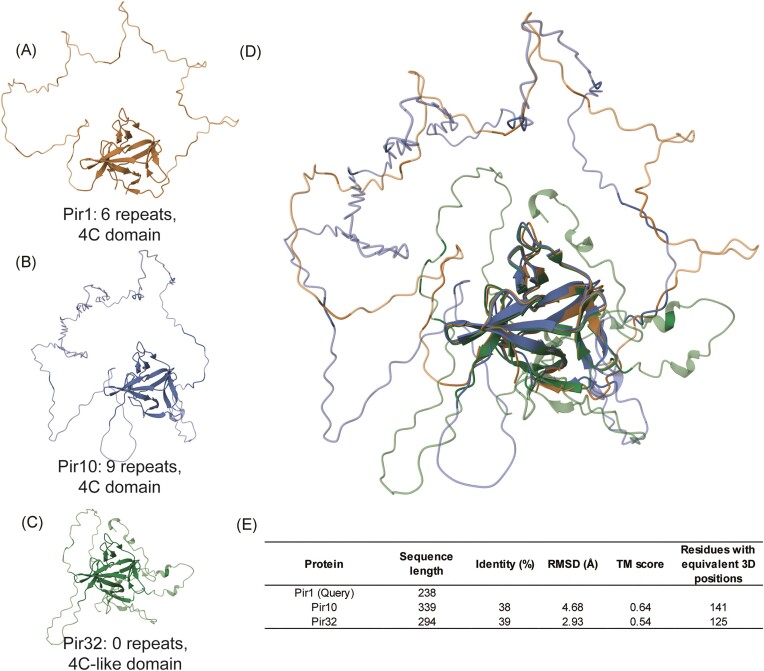
3D structural analysis of C. auris Pir proteins. (A–B) Pir1 paralogs harboring internal repeats and the conserved 4C domain. (C) Pir32 paralog lacking repeats and harboring a modified 4C domain. (D) RCSB TM-alignment of the three proteins showing structural conservation of the 4C domain. (E) Details of sequence and tertiary structure similarity between Pir1 and the other two Pir family proteins.

Previous mass spectrometry-based proteomic studies in *S. cerevisiae* and *Candida* species have identified two types of covalently bound CWPs: the majority are GPI-modified proteins (described below) linked to β-1,6-glucan (De Groot et al. 2005). A minor second group lacks GPI anchor peptides and is connected to β-1,3-glucan through alkali-sensitive linkages (ASL) (De Groot et al. 2005). Identified ASL wall proteins in baker’s yeast and *Candida* spp. include Pir, Bgl2, Sun, and Tos1 family members (de Groot et al. 2004, Yin et al. 2005, De Groot et al. 2008, Klis et al. 2009). Blast analysis confirmed that paralogs of the encoding genes are also present in the genomes of *C. auris* and the *C. haemuli*-complex species (Table 1 and Table S1). Linkage of *S. cerevisiae* Cis3/Pir4 to β-1,3-glucan has been shown to be achieved through the conserved glutamine-rich Pir-specific repeat (Ecker et al. 2006). This led to the hypothesis that Pir proteins with multiple repeats might crosslink β-1,3-glucans to reinforce the wall structure. Blast searches with Pir protein queries identified three paralogs in *C. auris* and the *C. haemuli*-complex species. Two are canonical preproproteins with multiple repeats and a 4C domain like the Pir proteins in *S. cerevisiae* (De Groot et al. 2005). The third contains a structurally conserved, slightly modified, 4C domain but lacks a Kex2 cleavage site and Pir repeats and, therefore, is more likely to be secreted than incorporated into the cell wall (Fig. 2 and Table S2).

**Figure 3.**
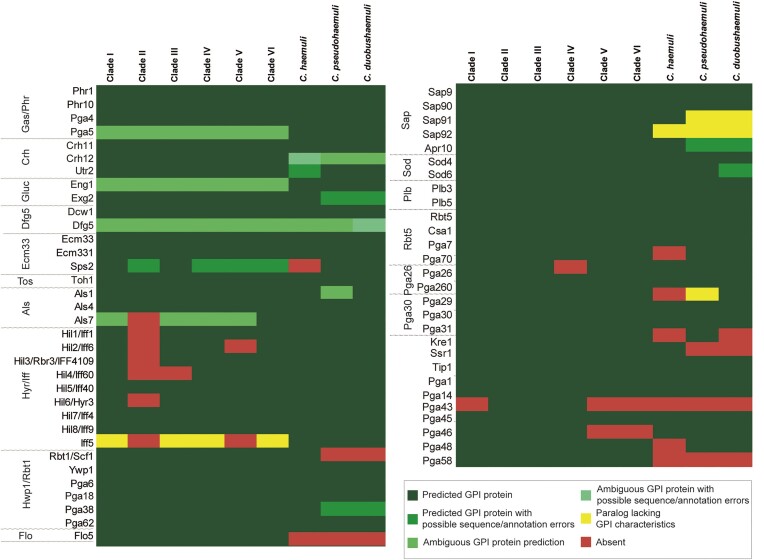
Conserved putative GPI proteins in the six *C. auris* clades and in *C. haemuli* complex species. GPI proteins were identified *in silico* and annotated according to their closest homologs in *C. albicans* or *S. cerevisiae* Flo5, as detailed in the “Materials and Methods” section.

For the identification of putative GPI proteins, we employed an earlier described bioinformatic pipeline (detailed in the “Materials and Methods” section), which in essence selects from translated genomes proteins that share three characteristics: (i) presence of an N-terminal signal peptide for secretion, (ii) presence of a C-terminal GPI-anchoring signal, and (iii) absence of transmembrane domains in the mature protein. As the pipeline depends on correct open reading frame (ORF) calling and curation in genome sequencing projects, Blast cross-checking of identified proteins among the different strains was performed to avoid any omission. The number of putative GPI proteins identified in the six *C. auris* clades were: 84 (clade I), 72 (clade II), 84 (clade III), 82 (clade IV), 80 (clade V), and 81 (clade VI) (Tables S3 and S4). In the *C. haemuli* complex species, we identified 76, 70, and 70 putative GPI proteins in *C. haemuli, C. pseudohaemuli*, and *C. duobushaemuli*, respectively (Table S5). The number of GPI proteins is similar to pathogenic CTG-clade *Candida* spp. (De Groot et al. 2003, Richard and Plaine 2007, Butler et al. 2009). Altogether, 88 putative GPI proteins were identified in *C. auris*, and for 61 of these subsequent Blast analyses indicated that they were homologs of known GPI proteins in *C. albicans* (Fig. 3 and Table S3), including Gas, Crh, Ecm33, Dfg5, adhesin (Iff, Als, and Hwp1/Rbt1), aspartyl protease, and phospholipase B protein family members. In addition, one protein without homology to *C. albicans* GPI proteins showed similarity to *S. cerevisiae* Flo-type flocculins, detailed below.

**Figure 4.**
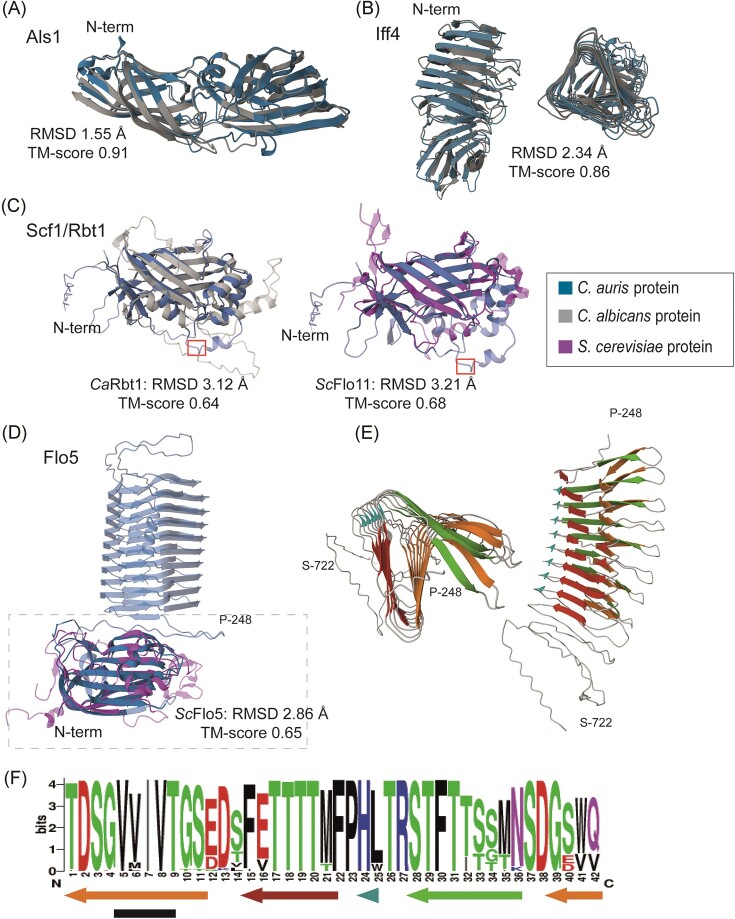
Comparative structural analysis of *C. auris* adhesins. (A–D) Cartoon presentations of modeled three-dimensional structures (AlphaFold2) of putative ligand-binding domains of *C. auris* adhesins aligned (RCSB TM-alignment) with their closest *C. albicans* or *S. cerevisiae* homologs. (A) Als family protein Als1; (B) Iff/Hyr family protein Iff4, side and top views; and (C) Scf1/Rbt1. A canonical surface-exposed Kex2 cleavage site (KR/DV) at positions 216–219 in Scf1 is indicated by a red box. (D) Flo5 (strain B11220). The Flo5 model includes the repeat domain (top part) downstream of the ligand-binding domain (boxed), the latter aligned to ScFlo5. N-terminal residues in the structures of the *C. auris* proteins are indicated. (E) Top and side view of the strain B11220 Flo5 42-aa repeat domain (aa 248–722). (F) Sequence logo of the 42-aa repeat based on all Flo5 repeats in six representative strains (one per clade). Arrows indicate β-sheet forming regions, and the black bar marks a small region with high β-aggregation propensities according to TANGO. Ca, *C. albicans* and Sc, *S. cerevisiae*.

Concordant with other studies (Santana et al. 2023, Smoak et al. 2023, Pelletier et al. 2024, Wang et al. 2024), among the identified GPI proteins are three ORFs showing similarity to Als and nine showing similarity to Hyr/Iff adhesins in *C. albicans* (Table 2 and Table  S3), which possesses eight and twelve members of these protein families, respectively. Despite the generally low levels of sequence identity of their putative ligand-binding domains with their closest homologs in *C. albicans*, AlphaFold2 modeling of their tertiary (3D) structures and subsequent structure similarity analysis showed low root mean square deviation (RMSD) and high template modeling (TM) scores (Fig. 4 and Table 2), confirming their identification as members of these adhesin families. With the exception of clade II, the distribution of these adhesin families among the different clades of *C. auris* was mostly conserved albeit that the *IFF* family in clades III and V appeared to have 1 or 2 gene copies less than clade I (Fig. 2). In clade II, various adhesin genes located in subtelomeric regions of other clades appeared to be absent due to chromosome rearrangements (Muñoz et al. 2021) (Fig. S1), reducing the *ALS* family to two and the *IFF* family to three gene copies.

**Table 2.** Identified adhesin-like proteins in *C. auris* strain VPCI479/P/13.

Protein	Length of LBD^a^	Identity of LBD with closest homolog (%)	RMSD (Å)	TM score	LBD residues with equivalent 3D positions
Iff1	305	38	1.75	0.87	281
Iff4	306	28	2.34	0.83	283
Iff40	307	34	2.13	0.85	282
Iff5	312	31	2.25	0.83	298
Iff6	308	33	1.81	0.85	282
Iff60	309	31	1.99	0.86	291
Iff9	310	33	2.12	0.85	284
Rbr3	305	36	1.79	0.86	282
Hyr3	308	33	2.03	0.86	286
Als1	313	30	1.55	0.91	305
Als3	308	33	1.60	0.92	299
Als7	302	27	1.91	0.91	294
Scf1/Rbt1	248	25 (*Ca*Rbt1)	3.12 (*Ca*Rbt1); 3.21 (*Sc*Flo11)	0.64 (*Ca*Rbt1); 0.68 (*Sc*Flo11)	169 (*Ca*Rbt1); 137 (*Sc*Flo11)
Flo5	193	31	2.86	0.65	193

a LBD, ligand-binding domain defined as the N-terminal part of the mature protein until the start of low complexity regions.

A third family of GPI proteins with adhesive functions in *C. albicans* is the Hwp1/Rbt1 family that includes the adhesins Hwp1, Hwp2, Eap1, and Rbt1, and Ywp1, a protein with proposed anti-adhesive properties (Granger et al. 2005, De Groot et al. 2013). These proteins do not show homology in their putative N-terminal ligand-binding domains, but they share the presence of (two copies of) a conserved 42-aa motif (De Groot et al. 2013). Among the putative GPI proteins in *C. auris* are proteins with the closest homology to Rbt1 and Ywp1. The Rbt1 paralog is better known as Scf1, an adhesin that was shown to play a key role in adherence to polystyrene (Santana et al. 2023). Its ligand-binding domain indeed shows structural properties resembling Rbt1 as well as *S. cerevisiae* Flo11, although it lacks primary sequence homology to the latter (Table 2 and Fig. 4). The 42-aa motif is also present in other *C. albicans* GPI proteins not predicted to be adhesins; Pga6, Pga18, Pga38, Pga59, and Pga62, the latter two being upregulated during biofilm formation (Moreno-Ruiz et al. 2009). *C. auris* contains homologs of Pga6, Pga18, Pga38, and Pga62 but only Pga38 presented the 42-aa motif as defined in *C. albicans*.

Another identified GPI protein, Flo5, showed (only) weak sequence similarity to *S. cerevisiae* flocculins Flo1, 5, 9, and 10 (Table 2). Structural relationship with these flocculins was supported by 3D modeling and subsequent alignment of their N-terminal ligand-binding domains (Fig. 4). Curiously, immediately downstream of the ligand-binding domain, Flo5 presents a variable number of a different type of 42-aa repeats, in the analyzed strains ranging from nine (clade II strains B11220 and B11809) to 97 copies (clade V strain IFRC2087) (Table  S6). In 3D structural models (Fig. 4), these repeat domains were displayed as channel-like structures of variable length, in which each repeat forms a helix turn that is predominantly composed of β-sheets, one with predicted β-aggregation propensities. To our knowledge, such a channel-like structure downstream of the ligand-binding domain has not been observed in any other fungal adhesin to date.

With respect to putative GPI proteins that are enzymes playing roles in tissue degradation or counteraction of host-defense responses, as earlier observed for *C. albicans*, our search identified aspartyl proteases, phospholipases, and superoxide dismutases in *C. auris* (Fig. 3). For four of the predicted GPI proteins, their closest homolog in *C. albicans* was the GPI-modified aspartic protease Sap9. Interestingly, another predicted *C. auris* GPI protein (Apr10) had higher identity with *C. albicans* Apr1, described to be a vacuolar proteinase, and not GPI-modified. The structural resemblance of Apr10 with Sap9-like proteases was supported by 3D modeling and alignment (Fig. S2). Ten other aspartic proteases with homology to non-GPI modified proteins in *C. albicans* lacked or had ambiguous GPI anchoring peptides (Table S7). Concerning phospholipase B proteins, two out of four were predicted to be GPI-modified in all analyzed strains by analogy to their closest homologs in *C. albicans* (Table S7). Strain B11245 uniquely had a third paralog (Plb4) with an ambiguous GPI anchoring prediction (Table S7). Other GPI proteins implicated in pathogenesis are GPI-anchored superoxide dismutases (Schatzman et al. 2020), the Rbt5 family mediating iron acquisition (Nasser et al. 2016), and Pga29/30/31- and Pga26-related proteins with demonstrated relevance for virulence but unresolved biological functions (de Boer et al. 2010, Laforet et al. 2011). All these families are present in *C. albicans* but absent in *S. cerevisiae*. Our analysis showed that paralogs were present in *C. auris* as well as the analyzed *C. haemuli* complex species (Fig. 3 and Table  S7).

**Figure 5.**
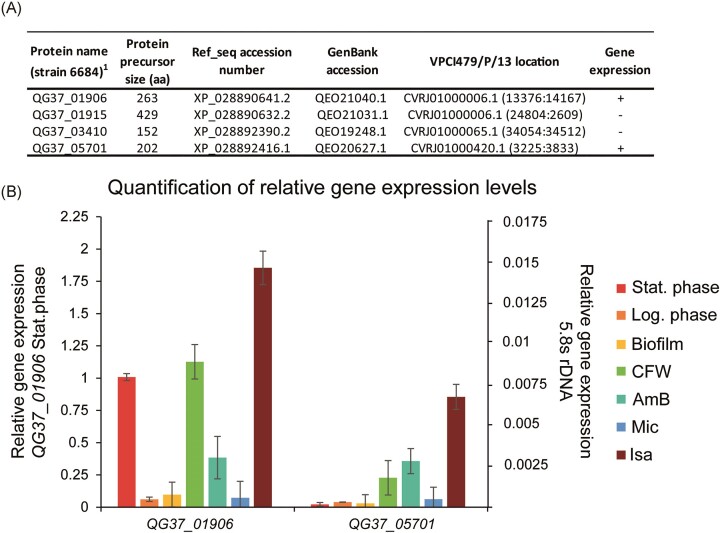
qPCR gene expression analysis of *C. auris* GPI protein-encoding genes lacking homologs in the *Candida* genus. (A) Four GPI protein-encoding genes were selected for gene expression analysis. (B) Relative gene expression levels of the two actively transcribed genes QG37_01 906 and QG37_05701. Data were normalized to 5.8S rDNA expression and plotted in comparison to QG37_01 906 (left axis) and 5.8S rDNA (right axis) transcript levels at the stationary phase. Shown are average values and standard deviations of two biological samples measured in triplicate.

Twenty-six of the identified putative *C. auris* GPI proteins lacked homologs in *C. albicans* or *S. cerevisiae* (Table S4 and Fig. S3). Twelve were detected in all *C. auris* clades. Of the proteins present in some but not in all *C. auris* strains, the majority were absent in clade II. Fifteen proteins were conserved in at least one of the *C. haemuli*-complex species. From a pathobiology point-of-view, cell surface proteins that are present in all *C. auris* clades but absent in the *Candida* genus are interesting candidates for further studies. For instance, their potential to serve as species-specific diagnostic PCR markers for *C. auris* infections has been demonstrated for some of the corresponding genes (Ruiz-Gaitán et al. 2018, Alvarado et al. 2021). Expression of four of such genes (one exclusively present in *C. auris*, one present in *C. auris* and *C. haemuli*, and two that are conserved in the *C. haemuli*-complex species) was analyzed by qPCR to explore their gene activity under infection-relevant conditions (Fig. 5). Conditions chosen for this analysis (detailed in the “Materials and Methods” section) were different growth phases (log phase, stationary phase, and biofilms), presence of subinhibitory concentrations of clinically used antifungals from different classes (AmB, Mic, and Isa) or the well-known cell wall perturbant CFW. The two genes that are conserved in the *C. haemuli* complex, *QG37_01906* and *QG37_05701*, were induced when grown in the presence of AmB, Isa, and CFW. The former was also expressed when grown to the stationary phase. The other two genes (*QG37_01915* and *QG37_03410*) were not expressed under any of the tested conditions (not shown) and were discarded for further functional analysis.

**Figure 6.**
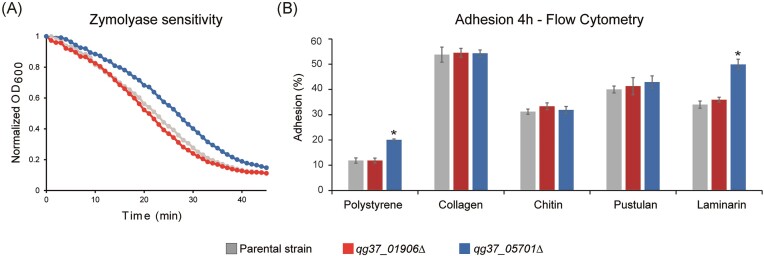
Deletion of QG37_05 701 but not QG37_01 906 affects glucan-related and adhesion properties of *C. auris*. (A) Zymolyase sensitivity. (B) Adhesion assays. Percentage of cells adhering to polystyrene (PS) and PS coated with different biomolecules after 4 h of incubation, measured by flow cytometry. Details concerning the number of replicates in each assay and statistical analysis are provided in the “Materials and Methods” section. Error bars indicate standard deviations. *P*-values < .05 (*) were considered statistically significant.

Deletion mutants were generated for the expressed GPI protein-encoding genes *QG37_01906* and *QG37_05701* in clade I strain VPCI479/P/13 to analyze their importance for fitness and cell surface-related properties. Deletion of neither of the two genes affected fitness in YPD cultures. The *qg37_01906*Δ mutants also did not show any phenotype in further assays that could provide evidence for a cell surface-related function. In the case of the *qg37_05701*Δ mutants, when testing sensitivity to cell wall (CFW, CR, and zymolyase) and membrane (SDS) perturbants and antifungal compounds (AmB, Flu, Isa, Cas, and Mic), only a slight decrease in sensitivity to zymolyase and a two-fold increase in sensitivity to Isa was observed (Fig. 6 and Fig. S4). The mutants did not have alterations in sedimentation, aggregation, or virulence in the *Galleria mellonella in vivo* model (not shown). The mutants also did not have phenotypes in assays measuring CSH, adhesion/biofilm formation onto polystyrene (PS) after 24 h of incubation, or adhesion to HeLa cells after 2 h of co-incubation (Fig. S4). Adhesion to PS and PS coated with different biomolecules was assayed by flow cytometry after 4 h of incubation (Fig. 6). In these assays, the *qg37_05701*Δ mutants showed increased adhesion to PS and laminarin (β-1,3-glucan)-coated PS but adhesion to PS coated with pustulan (β-1,6-glucan), chitin, or collagen was unaffected. Together with the decreased sensitivity to zymolyase, these data suggested that deletion of QG37_05 701 caused cell wall alterations related to β-1,3-glucan structure, amount, or accessibility.

## Discussion

The cell wall is an organelle that plays a key role in pathogen–host interactions and the establishment of fungal infections. In this paper, we present a detailed comparative genomic analysis of cell wall biosynthesis in *C. auris*, denoted as a “critical” emerging pathogenic yeast by the WHO (World Health Organization 2022). We achieved this through a combination of homology searching and the application of an independent bioinformatic pipeline to exhaustively identify the putative CWP-encoding genes. Our analysis included representatives of all six genetic *C. auris* clades as well as closely related species from the *C. haemuli* complex. All yeast species that are known to cause candidiasis, including *C. auris*, are categorized within the subphylum *Saccharomycotina* (Fig. 1A). We compared our data to the well-studied yeasts *C. albicans* (clinical pathogen, genus *Candida*) and *S. cerevisiae* (baker’s yeast, genus *Saccharomyces*). Importantly, our genomic analysis indicated that the cell wall architecture in *C. auris* and related species is built with the same macromolecules. However, alterations in protein families point to subtle differences, with *Candidozyma* species seemingly having a structure that is a hybrid of *Saccharomyces* and *Candida*, reflecting the relatively large phylogenetic distances between the three genera (Fig. 1A).

Results from BLAST searching indicated that, similar to *C. albicans* and *S. cerevisiae*, the cell wall in *C. auris* is composed of β-1,3-glucan, β-1,6-glucan, chitin, and mannoprotein, in accordance with literature that already documented the presence of these molecules (Bruno et al. 2020, Shivarathri et al. 2020, Farooqi et al. 2021, Wang et al. 2022). For all *C. albicans* and *S. cerevisiae* queries homologs were identified in *C. auris* and the related species. In a few cases, the different copy numbers of gene homologs point to differences in the amount, arrangement, or remodeling of the respective cell wall molecules. For instance, the Fks1 family encoding the catalytic subunit of the β-1,3-glucan synthase complex (Douglas 2001) is restricted to two genes in *Candidozyma* species (except *C. haemuli*) compared to three genes in *C. albicans* and *S. cerevisiae*. On the other hand, the Eng1-like endoglucanase family, implicated in β-1,3-glucan remodeling or degradation during cytokinesis (Esteban et al. 2005), is expanded by one gene in the *Candidozyma* species. As for protein mannosylation, noteworthy differences were identified for the Mnn4 and Bmt families involved in phosphomannosylation. Regarding the Mnn4 family regulating mannosylphosphate addition (Hall and Gow 2013), *C. albicans* comprises eight copies, *S. cerevisiae* has only two, and the *Candidozyma* species present an intermediate situation with six gene copies. Similarly, where *C. albicans* performs β-mannosylation with nine *BMT* gene copies (Hall and Gow 2013), *S. cerevisiae* lacks this gene family, however, *C. auris* and the other analyzed *Candidozyma* species possess two *BMT* genes, suggesting modest β-mannosylation (Fig. 1B). These examples emphasize the phylogenetic distances between the three different genera (Fig. 1A). Although we should be very careful to extrapolate *in silico* data to actual amounts of cell wall molecules, the lowered number of *MNN4* and *BMT* gene copies in *C. auris* seems consistent with NMR studies indicating lowered levels of phosphomannan and β-mannosylation compared to *C. albicans* (Wang et al. 2022). On the other hand, our analysis does not provide hints pointing to a higher level of total cell wall mannan in *C. auris* as measured by HPLC and gas chromatography analysis in the same publication.

An interesting difference between the three genera is also apparent in the Pir family of mild-alkali-sensitive CWPs. Pir proteins are incorporated in the wall through a direct ester link between a glutamine residue in their typical Pir repeats and β-1,3-glucan, as elegantly shown for *S. cerevisiae* Cis3/Pir4 (Ecker et al. 2006). Owing to the presence of multiple Pir repeats in most of these proteins, they are hypothesized to strengthen the cell wall by β-1,3-glucan crosslinking. In *S. cerevisiae* (and *C. glabrata*), the Pir family is encoded by five gene copies, while a few other—GPI-modified—CWPs also contain a single Pir repeat sequence, suggesting possible β-1,3/β-1,6-glucan crosslinking (De Groot et al. 2008). Apart from the typical repeats, Pir proteins also share a C-terminal fold supported by four cysteines at conserved positions. *C. albicans* has two proteins, Pir1 and Pir32, with a similar fold, however, Pir1 is the only protein that contains Pir repeats and has been identified in cell wall preparations (Heilmann et al. 2011, Sorgo et al. 2011). Therefore, considering their proposed role in cell wall reinforcement, it may seem surprising that neither deletion of *PIR1* nor simultaneous deletion of *PIR1* and *PIR32* led to cell wall or fitness defects (Kim et al. 2022). This suggests that *C. albicans* may have developed other mechanisms to achieve sufficient cell wall strength. In the analyzed *Candidozyma* species, three proteins with the C-terminal Pir fold were present (Fig. 2), two of which have internal repeats and are therefore likely to be incorporated into the cell wall (Fig. 1B). The third has closer homology to Pir32 and is more likely to be secreted. Hybrid proteins with GPI anchor peptides and a Pir repeat are absent in *C. auris* and related species.

Previous genomic studies have indicated that the number of GPI proteins in fungal species seems to reflect their pathogenicity (Butler et al. 2009). Also in this case, compared to *C. albicans* (106 GPI proteins) and *S. cerevisiae* (~70), *C. auris* presents an intermediate situation with the number of GPI proteins ranging from 84 (clades I and III) to 72 (clade II). Interestingly, clade II is considered less pathogenic and lacks various adhesin genes in telomeric regions (Muñoz et al. 2021) (Fig. S1). This resembles the observed enrichment of adhesin families Hyr/Iff and Als in the more pathogenic *Candida* spp. in the order *Serinales* compared to less pathogenic species (Butler et al. 2009). In line with this, in the other analyzed *C. haemuli* complex species, that are less pathogenic, the number of identified GPI proteins (*Ch*, 76; *Cp*, 70; and *Cd*, 70) is lower than in *C. auris* (except clade II) and is closer to the number in baker’s yeast.

*Candida auris* also presents an intermediate situation when comparing cell wall adhesins with *C. albicans* and *S. cerevisiae. C. albicans* has three adhesin families, Als (8 genes), Iff (12), and Hwp1/Rbt1 (10), whereas *S. cerevisiae* has two sexual agglutinins and Flo1, 5, 9, 10, and 11 flocculins. In *C. auris*, consistent with earlier reports (Santana et al. 2023, Smoak et al. 2023), our GPI pipeline identified three Als and nine Hyr/Iff proteins. Of the latter family, Iff4109/Rbr3, has been shown to mediate adhesion and colonization of inert surfaces and mammalian hosts (Santana et al. 2023). In addition, another putative GPI adhesin, Scf1/Rbt1, has structural similarity to both *Ca*Rbt1 and *Sc*Flo11, supporting the previously hypothesized functional link between these adhesins (Monniot et al. 2013). Scf1/Rbt1 was shown to mediate colonization of skin and inserted medical devices, biofilm formation, and virulence in systemic infection, relying on exposed cationic residues for surface association (Santana et al. 2023). Scf1-mediated adhesion to polystyrene is governed, at least partly, by a cation-aromatic amino acid cluster in the N-terminal domain (Santana et al. 2023). Intriguingly, downstream of the N-terminal domain, the protein contains at least one surface-exposed canonical substrate site for Kex2 cleavage (Bader et al. 2008) (Fig. 4), which might result in secretion of the N-terminal functional domain as (a) soluble peptide(s) rather than being linked to the cell wall. This would be consistent with the fact that *SCF1* deletion does not affect CSH and that an Scf1 protein carrying a FLAG-tag immediately downstream of the N-terminal domain localizes to the cell surface (Santana et al. 2023). If true, an important question may be how a soluble secreted protein domain mediates in abiotic and cell surface adhesion leading to biofilm formation. Consistent with the study of Santana and co-workers (Santana et al. 2023), our pipeline also detected a homologous GPI protein in *C. haemuli* but not in *C. pseudohaemuli* or *C. duobushaemuli*. Finally, a putative adhesin (Flo5) with structural similarity to the N-terminal domain of *S. cerevisiae* Flo1,5,9 and 10 was only identified in *C. auris*. Interestingly, in contrast to the *S. cerevisiae* paralogs, Flo5 contains a channel-like helix structure composed of (a variable number of) 42-aa repeats immediately downstream of the N-terminal domain. This domain has predicted β-aggregation propensities and therefore might promote formation of amyloids and flocculation (Golan et al. 2022). The presence of both adhesins typical for *C. albicans* and for *S. cerevisiae* is not unique for *C. auris* as it has previously also been documented for *C. krusei* (Alvarado et al. 2023) and *C. glabrata* (Reithofer et al. 2021, Smoak et al. 2023); it seems to reflect that all these species belong to different genera in the same subphylum (Fig. 1A).

GPI-modified aspartic proteases and phospholipase B proteins have roles in pathogenesis (Klis et al. 2009). Although these proteins are conserved in the genomes of analyzed *Saccharomycotina*, aspartic proteases have only been identified in cell wall preparations of some pathogenic *Candida* species (Heilmann et al. 2011, Moreno-Martinez et al. 2021, Alvarado et al. 2023). Other families of GPI proteins implicated in pathogenesis include superoxide dismutases, proteins mediating iron acquisition, and Pga29/30/31- and Pga26-related proteins. Except perhaps Pga26, these proteins are conserved among the more pathogenic species in the *Candida* genus but are absent in *Saccharomyces* (Butler et al. 2009). Concordant with their pathogenic potential, our pipeline identified all these GPI protein families in *C. auris* as well as in the *C. haemuli*-complex species.

Although GPI anchoring is a feature that is present in all eukaryotes, GPI-modified CWPs are not known to be essential and are relatively less conserved. Previous genomic studies on *Debaryomycetaceae* yeasts indicated that each contains a small number of species-specific GPI-modified singletons [varying between nine and 26, (Butler et al. 2009)]. This is also the case for *C. auris* where 11 GPI-modified singleton genes were identified. Consistent with the poor conservation of GPI proteins, only four of these were detected in all six clades, although we cannot exclude that this is partly due to experimental errors. For 15 others a homolog was discovered in at least one of the *C. haemuli* complex species but not in the *Debaryomycetaceae* family. These species-specific proteins are promising candidates for developing efficient novel antifungal strategies, including diagnostic markers, antifungal compounds, or vaccines against this hazardous species in addition to more generic pan-*Candida* or panfungal approaches (Gow et al. 2017, Cortes et al. 2019). Furthermore, genes that are uniquely present in a pathogen like *C. auris* may also be relevant for its pathobiology. Of the two genes that were studied here, deletion mutants lacking *QG37_05701* showed mild phenotypes related to β-1,3-glucan properties consistent with its predicted cell surface localization. If this is somehow related to its pathogenicity remains to be elucidated.

In conclusion, our genomic study indicates that cell wall construction in the emerging pathogen *C. auris* is based on the same principles as described for baker’s yeast and *C. albicans*. However, expansions and reductions in the implicated protein families point to subtle but remarkable differences, hinting to intermediate cell wall aspects, for instance, regarding β-1,3-glucan, protein mannosylation, number of GPI proteins, and types of adhesins. Altogether, our study uncovered a wealth of genomic information on cell wall synthesis in *C. auris*. This may serve future studies aimed at improving our understanding of the relationship between cell wall structure and pathogenicity and at the development of novel antifungal approaches.

## Supplementary Material

foae039_Supplemental_Files
