# Supplementary figures and images for: The good, the bad, and the hazardous: comparative genomic analysis unveils cell wall features in the pathogen Candidozyma auris typical for both baker’s yeast and Candida
Source: FEMS Yeast Res. 2024 Dec 4;24:foae039. doi: 10.1093/femsyr/foae039 (PMC11657238; doi:10.1093/femsyr/foae039)

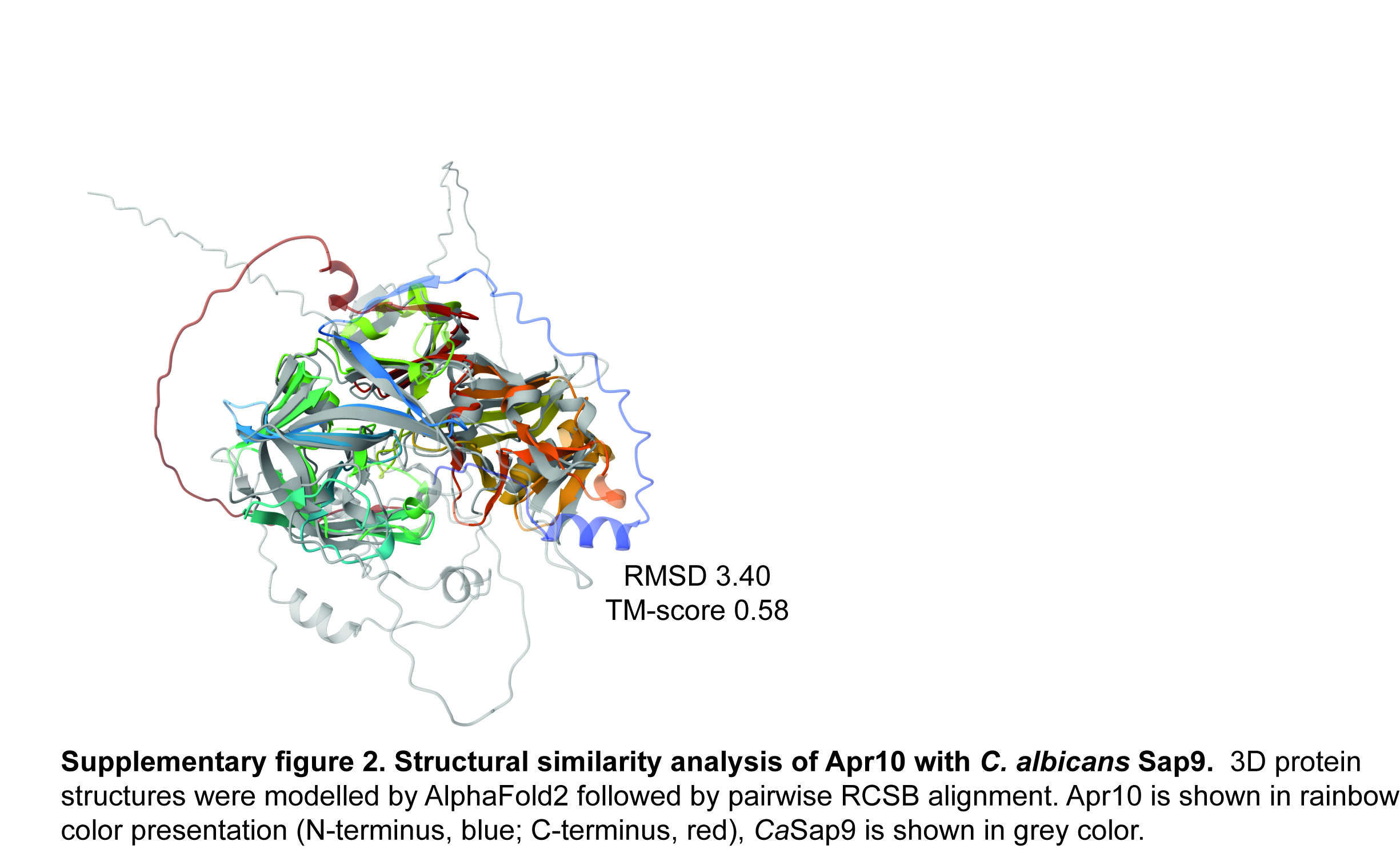

Supplement: foae039_Supplemental_Files [file foae039_supplemental_files.zip › S2.tif]

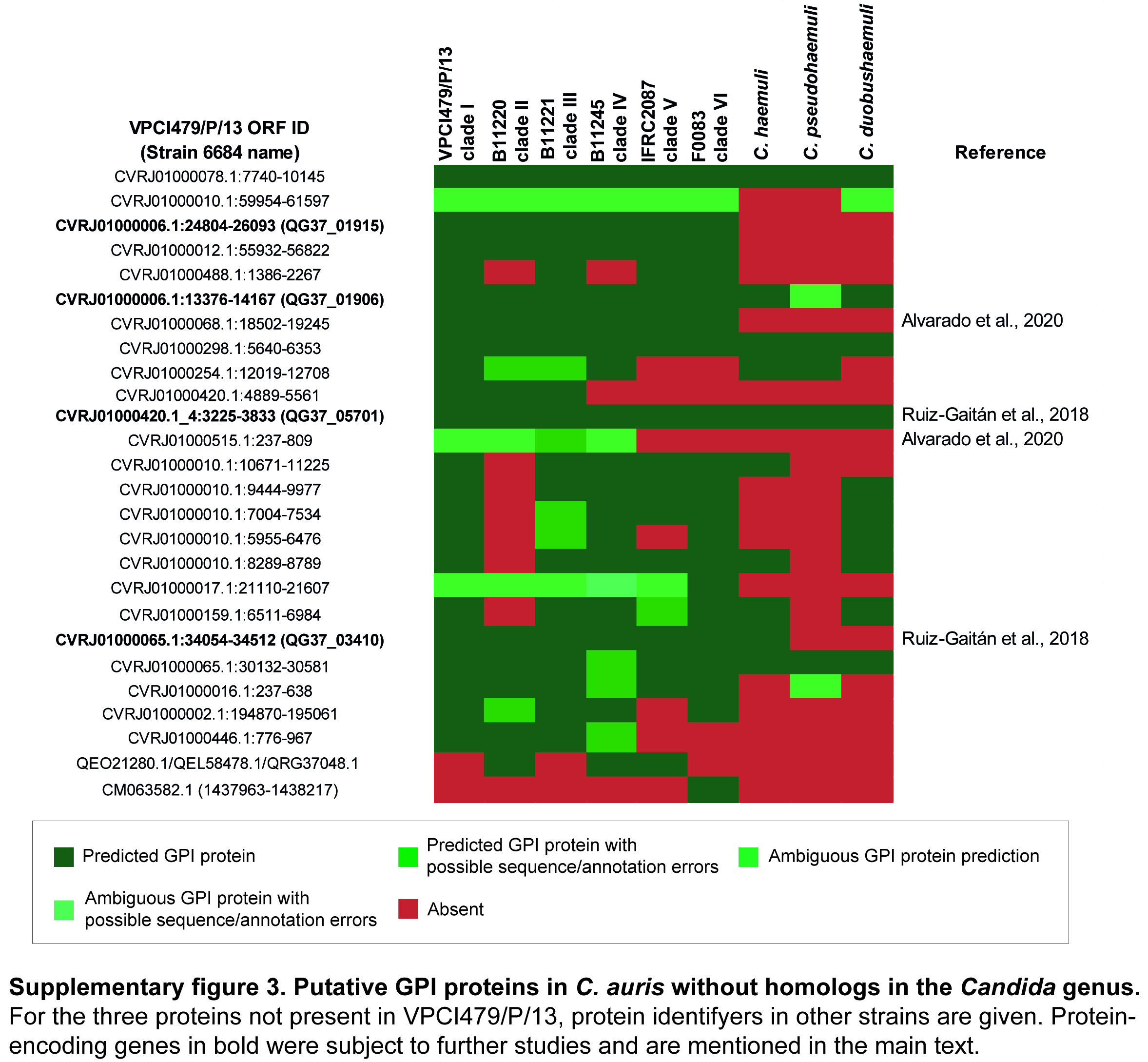

Supplement: foae039_Supplemental_Files [file foae039_supplemental_files.zip › S3.tif]

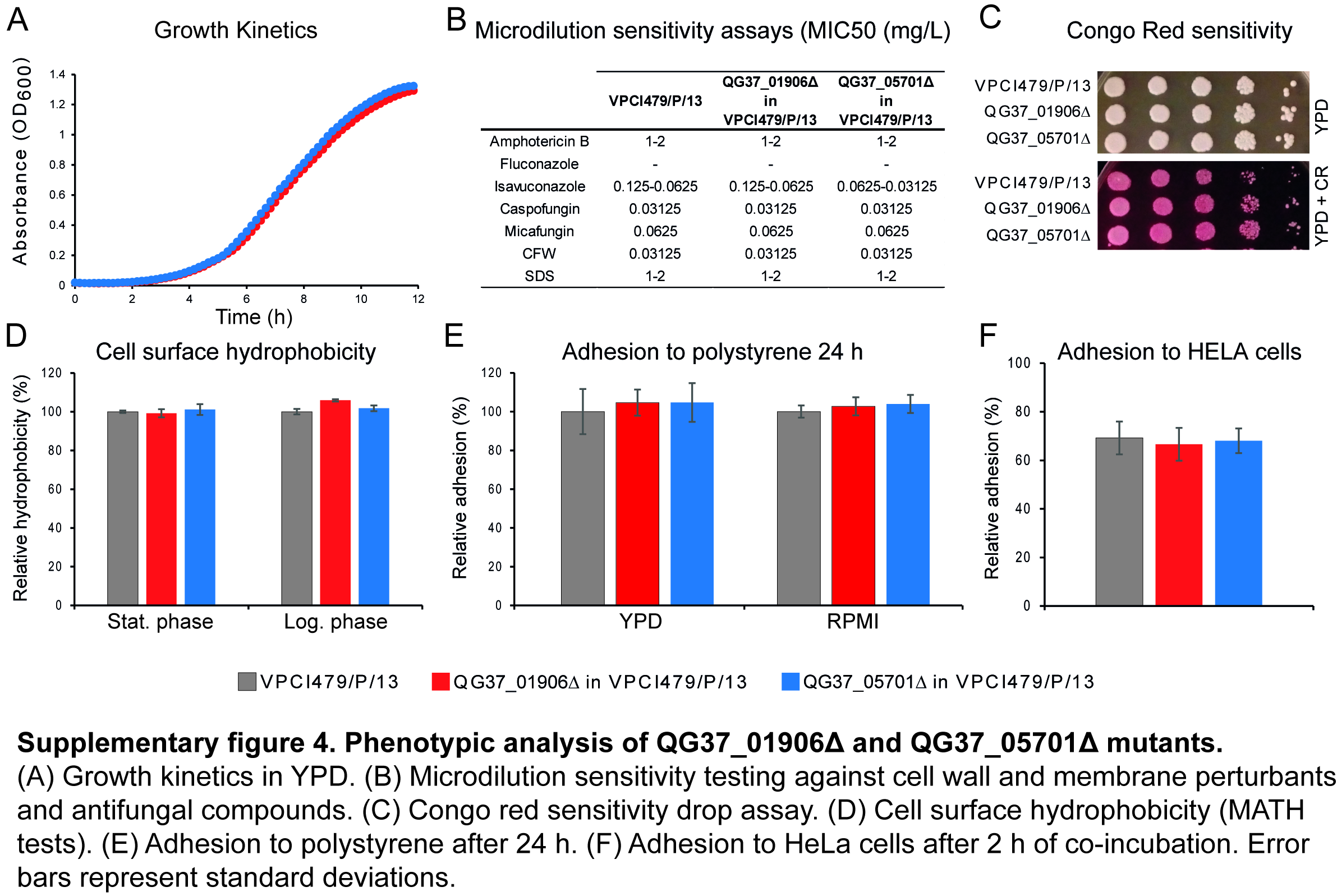

Supplement: foae039_Supplemental_Files [file foae039_supplemental_files.zip › S4.tif]
